# Estimation of Prevalence of Hospital-Acquired Blood Infections among Patients Admitted at a Tertiary Hospital in Oman over a Period of Five Years: A Cross-Sectional Study

**DOI:** 10.1155/2023/5853779

**Published:** 2023-05-08

**Authors:** Marah El-Beeli, Yahya Al-Farsi, Abdullah Balkhair, Zakariya Al-Muharrmi, Mansoor Al-Jabri, Samir Al-Adawi

**Affiliations:** ^1^Department of Family Medicine and Public Health, College of Medicine and Health Sciences, Sultan Qaboos University, Muscat, Oman; ^2^Department of Epidemiology, School of Public Health, Boston University, Boston, Massachusetts, USA; ^3^Department of Infection Control, Sultan Qaboos University Hospital, Sultan Qaboos University, Muscat, Oman; ^4^Department of Behavioral Medicine, College of Medicine and Health Sciences, Sultan Qaboos University, Muscat, Oman

## Abstract

**Background:**

Data from developed/developed countries have shown that hospital-acquired blood infections (HA-BSIs) are one of the most severe nosocomial infections and constitute 20%–60% of hospitalization-related deaths. Despite the high morbidity and mortality rates and the enormous burden of health care costs associated with HA-BSIs, to our knowledge, there are few published reports on HA-BSI prevalence estimates in Arab countries, including Oman.

**Objectives:**

This study aims to explore the HA-BSI prevalence estimates over selected sociodemographic characteristics among admitted patients at a tertiary hospital in Oman over five years of follow-up. The regional variations in Oman were also examined in this study.

**Methods:**

This hospital-based cross-sectional study reviewed reports of hospital admissions over 5 years of retrospective follow-ups at a tertiary hospital in Oman. HA-BSI prevalence estimates were calculated over age, gender, governorate, and follow-up time.

**Results:**

In total, 1,246 HA-BSI cases were enumerated among a total of 139,683 admissions, yielding an overall HA-BSI prevalence estimate of 8.9 cases per 1000 admissions (95% CI: 8.4, 9.4). HA-BSI prevalence was higher among males compared to females (9.3 vs. 8.5). HA-BSI prevalence started as relatively high in the group aged 15 years or less (10.0; 95% CI 9.0, 11.2) and then declined as age increased from 36 to 45 years (7.0; 95% CI 5.9, 8.3) when it started to increase steadily with increasing age in the group aged 76 or more (9.9; 95% CI 8.1, 12.1). The governorate-specific estimate of HA-BSI prevalence was the highest among admitted patients who resided in Dhofar governorate, while the lowest estimate was reported from the Buraimi governorate (5.3).

**Conclusion:**

The study provides supportive evidence for a steady increase in HA-BSI prevalence over age categories and years of follow-up. The study calls for the timely formulation and adoption of national HA-BSI screening and management programs centered on surveillance systems based on real-time analytics and machine learning.

## 1. Introduction

Hospital-acquired bloodstream infections (HA-BSIs) are one of the most severe hospital-acquired infections (HAIs) [1–3]. Surveys have shown that HA-BSIs were responsible for 20%–60% of hospitalization-related deaths [4, 5]. In the United States, HA-BSIs represent 10% of HAIs and have been ranked as the eleventh leading cause of death, with 99,000 deaths annually from 1.7 million, who have contracted HAIs [6]. In some Western European countries, HA-BSIs have been established to trigger high morbidity rates and two-thirds of the annual deaths [7].

Although HA-BSIs are likely to thrive in various geographical and ethnicities around the world, the prevalence of HA-BSIs has been generally reported from the industrialized countries of Northern America, Western Europe, and pocket countries in the Pacific Rim. It is, therefore, necessary to conduct studies that have documented HA-BSIs in various parts of the world, so that the best practices can be considered globally. There is an urgent need to document the pattern of HA-BSIs in emerging economies since most of the global population concentrates on developing countries. Some preliminary data indicate that, in addition to the dearth of studies, those regions appear to have suboptimal patient safety measures, including infection control practices [8, 9]. Studies by authors in [10, 11] indicated rampant compliance with safety measures. Due to limited resources and safety culture [9], the prevailing health situation in developing countries would appear bleak in the foreseeable future [10]. Therefore, studies on patient safety, including hospital-acquired infections, are needed.

Studies addressing the burden of hospital-acquired infections, including the prevalence of HA-BSIs and its microbial pathogens in developing countries, are critical to contemplate preventive measures. Among the developing countries in the Middle East and North Africa, some studies have started to quantify the burden of BSI [8, 10, 12, 13]. In the Gulf Cooperation Council (GCC), some studies have emerged on various aspects of HA-BSIs [11, 14–18].

To our knowledge, in Oman, previous reports have suggested that HA-BSIs may be prevalent in Oman, including multidrug-resistant organisms [19–24]. This study aims to explore (i) the sociodemographic characteristics of the patients who have had HA-BSI and (ii) the prevalence of HA-BSI, and the regional distribution and pathogens associated with the HA-BSI.

## 2. Methods

### 2.1. Study Design

To achieve the stated objectives, an ambidirectional, hospital-based, cross-sectional study of HA-BSIs has been undertaken at Sultan Qaboos University Hospital (SQUH) for five years, from January 2015 to 2019. SQUH is a 570-bed tertiary care teaching hospital in Muscat, Oman, receiving its referrals from regional, secondary, and primary healthcare centers in seven governorates (i.e., Muscat, Batinah, Dakhiliyah, Sharqiya, Dhofar, Dhahirah, and Buraimi). The hospital includes an Infection Control Unit consisting of doctors and nurses specialized in infection control-related disciplines.

### 2.2. Case Definition and Ascertainment

The CDC definition and classification of BSI and HA-BSI were adopted [25]. Therefore, BSI was diagnosed by one positive blood culture, if the isolate is a recognized pathogen, one blood culture from the central line, or at least two blood cultures from the peripheral line if the isolate is one of the common commensals. BSI cases were classified as hospital-acquired cases if the laboratory-confirmed bloodstream infection occurs after 48 hours or more of hospital admission. The study included all adults and children, patients, and newborns with HA-BSI during the study period. Newborns with HA-BSI less than 48 hours of delivery were also included in the study. The situation in which BSI occurred due to earlier admission, and the patient was discharged not more than 48 hours after the last admission, was also considered as HA-BSI.

### 2.3. Data Collection

The data were collected by reviewing patient medical records on the hospital information system (HIS). The data collected included sociodemographic data, clinical information, and microbiological information. Two clinical investigators developed and employed a coding guide based on the International Statistical Classification of Diseases and Related Health Problems (ICD) criteria. They reviewed and scored all evaluations to determine if the HA-BSI labeling was consistent with the standard international diagnostic criteria of BSI.

The inter-rater reliability among the two clinical investigators culminated in 94% agreement on the overall HA-BSI case status. For ongoing inter-rater reliability checks, a random sample of records (10%) was scored independently by a reviewer with clinical experience in the management of HA-BSI and did not participate in the diagnostic reviews. The percentage agreement between the raters on the final HA-BSI case definition was 96%.

### 2.4. Statistical Analysis

A general analysis strategy has been formulated to follow which was described by Alsumait et al. [26] and Nguyen [27]. The prevalence of HA-BSI in patients was calculated by dividing the number of HA-BSI cases by the total admission. The prevalence of HA-BSI isolates was calculated by dividing the number of HA-BSI isolates by the total admission. Prevalence was reported per 1,000 admissions. The 95% confidence intervals (95% CI) of prevalence estimates were calculated using the Poisson distribution method of binomial variables. A *P* value of 0.05 or less was considered statistically significant. Statistical Package for Social Sciences (SPSS) (version 24.0, IBM) was used for all statistical analyses. Ethical approval for this study was obtained from the Medical Research Ethics Committee at the College of Medicine and Health Sciences, Sultan Qaboos University.

## 3. Results

Table 1 shows the distribution of the selected sociodemographic and clinical characteristics of admissions enrolled in the study. There were 139,683 admissions, of which 1246 (0.89%) were HA-BSI cases. Admitted male patients were slightly higher (71,823; 51.4%). More than half (58.6%) of the admissions were patients aged below 45. Over the years, there has generally been a steady distribution of total admissions. Most admitted patients (34.7%) were residents of Muscat Governorate, followed by Batinah and Dakhiliyah (18.5% and 13.7%, respectively).

Table 1 also compares the selected characteristics among HA-BSI cases versus noncases. The gender distribution was similar around 50% among both groups. HA-BSI cases tended to be younger in age. The distributions of each governorate of residence and year of procedure request were comparable among HA-BSI cases and noncases. In all comparisons, the differences were not statistically significant (*p* > 0.05).

### 3.1. Prevalence Rates of HA-BSI in Patients and Isolates

**Figure 1:**
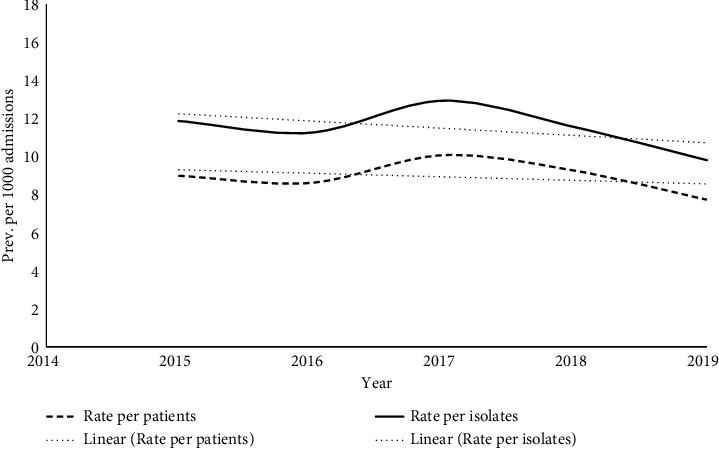
Prevalence estimates of HA-BSI among admitted patients and in isolates, stratified by year of admission, Oman.

Table 2 and Figure 1 show the prevalence of HA-BSI among admitted patients and in isolates, by the year of admission. The overall prevalence of HA-BSI among admitted patients was 8.9 (95% CI 8.4, 9.4) cases per 1,000 admissions, while the overall prevalence of HA-BSI among isolates was 11.5 cases (95% CI 10.9, 11.0) per 1,000 admissions. Overall, there has been a slight decrease in the prevalence of HA-BSI patients and isolates during the period from 2015 to 2019. In addition, the prevalence estimates of the isolates were greater than those of the cases over the years of admission.

**Figure 2:**
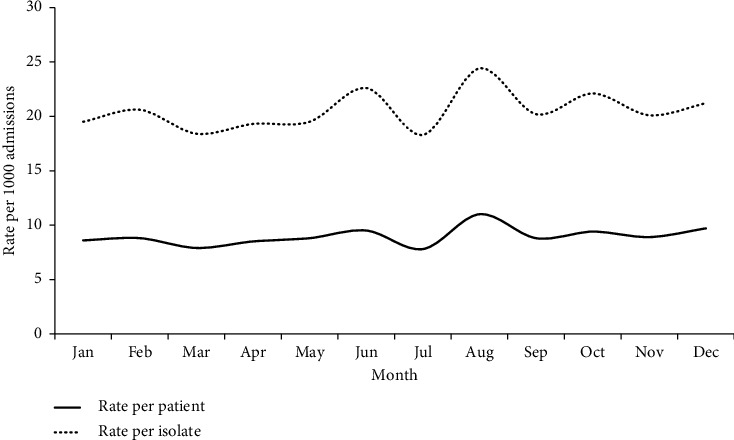
Prevalence estimates of HA-BSI among admitted patients and in isolates, stratified by the month of admission, Oman.

Table 3 and Figure 2 illustrate the seasonal trend of the occurrence of HA-BSI throughout the year, stratified by the month of admission. The prevalence of HA-BSI cases and isolates was steady from January to May. In June, the prevalence increased and continued to fluctuate until the end of the year. The highest prevalence was observed in August (11.0; 95% CI 9.1, 13.2), while the lowest prevalence was observed in July (7.8; 95% CI 6.4, 9.5).

### 3.2. Age-Specific Prevalence Estimates

**Figure 3:**
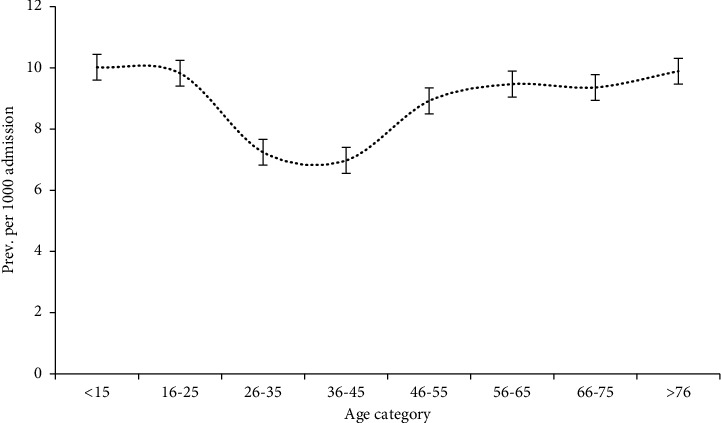
Distribution of HA-BSI prevalence estimates over age categories, Oman.

Table 4 shows the frequency and prevalence estimates of HA-BSIs among admitted patients stratified by age and gender. The HA-BSI prevalence per 1000 admissions among males (9.3; 95% CI 8.6, 10.0) was higher than that among females (8.5; 95% CI 7.9, 9.3). The age-specific prevalence estimates indicated that the HA-BSI prevalence estimates started relatively high in the group aged 15 years or less (10.0; 95% CI 9.0, 11.2), and then declined as age increased from 36 to 45 years (7.0; 95% CI 5.9, 8.3) then it started to increase steadily with increasing age in the group aged 76 or more (9.9; 95% CI 8.1, 12.1).  Figure 3 depicts the distribution of HA-BSI prevalence estimates over increasing age categories.

**Figure 4:**
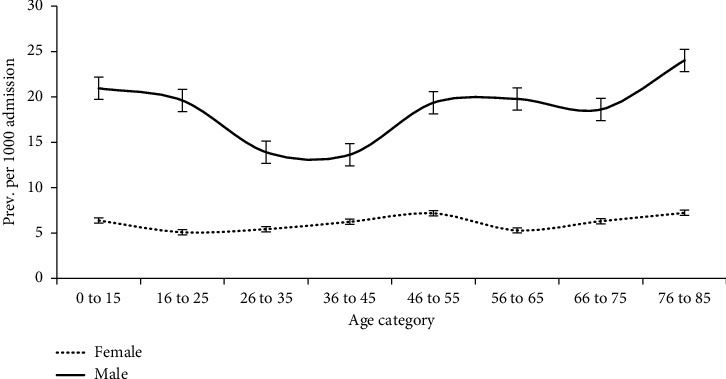
Distribution of gender-specific HA-BSI prevalence estimates over age categories, Oman.

Figure 4 depicts the distribution of gender-specific prevalence estimates over increasing age categories. HA-BSI prevalence among males was proportionately higher than that among females in all age categories. HA-BSI prevalence among males started high among patients aged 15 years or less. It then declined gradually till the age category 36 to 45 years , and then it started to increase steadily, reaching the highest estimate among people aged 70 or older. The HA-BSI prevalence among females appears to follow a steadier pattern over all age categories.

As shown in Table 5, the highest prevalence recorded was in the ICU ward at 43.5 (95% CI 39.0, 48.6). Among the total of 6,915 admissions in the ICU over five years, there have been 301 HA-BSI cases. Medical wards came second after the ICU at 16.9 (95% CI 15.5, 18.4), followed by child health and surgical wards at 8.4 (95% CI 7.4, 9.4) and 3.8 (95% CI 3.2, 4.5), respectively. The lowest prevalence was obtained from OBGYN (0.5; 95% CI 0.3, 0.8).

Table 6 illustrates that most HA-BSI patients were from the medical wards, where the percentage ranged from 38.2% in 2015 to 45.8% in 2017. ICU comes next with an overall percentage of 24.2%. HA-BSI among patients at OBGYN wards was only 1.4%. It has been observed that the prevalence rate of HA-BSI significantly increased in the ICU ward (70.2 (95% CI 56.3, 87.2)) in 2017. Moreover, the highest record prevalence rate of HA-BSI in the medical ward was in 2017 (20.0 (95% CI 16.8, 23.8).

**Figure 5:**
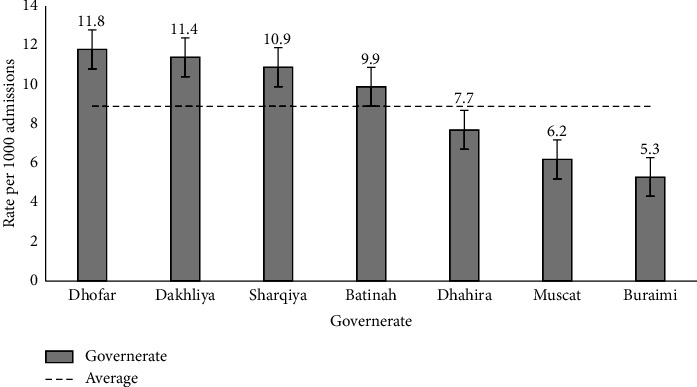
Governorate-specific prevalence estimates of HA-BSI among admitted patients, Oman.

Table 7 and Figure 5 show the governorate-specific estimates of HA-BSI prevalence among admitted patients. The highest prevalence estimate per 1,000 admissions was reported among admitted patients who resided in the Dhofar governorate (11.8; 95% CI 10.2, 13.8), followed by Dakhiliyah (11.4), Sharqiyah (10.9), Batinah (9.9), Dhahirah (7.7), and Muscat (6.2). The lowest prevalence estimate was reported from the Buraimi governorate (5.3; 95% CI 3.5, 9.4).

## 4. Discussion

In a hospital setting, the presence of immune-compromised patients, the lack of vigilance to hygiene, and the inherent tendency of medical and surgical procedures to encroach on natural protective barriers in the human body all can trigger the proliferation of spreading pathogens. Some developed countries have established infection control strategies with nationwide concurrent surveillance studies, which, in turn, have generally mitigated the vagary of nosocomial infections. Some hospitals in some Western countries have instituted pay or retribution to healthcare settings for their vigilance in confronting nosocomial infections. There needs to be more evidence to suggest that such preventive measures are widespread in developing countries such as Oman.

This study was performed to determine the prevalence of HA-BSI in a tertiary care hospital in Oman. The overall prevalence rates of HA-BSI per patient and isolates were 8.9 per 1000 admissions and 11.5 per 1000 admissions, respectively. Similar studies were conducted in the USA and Europe, and slightly lower readings were obtained [28]. A Chinese surveillance study reported a prevalence rate of 5.7/1000 admissions in a traditional Chinese medicine hospital [29]. Our prevalence rate mimics the results obtained from other studies conducted in other developing countries such as Saudi Arabia, Jordan, and Qatar (8.5, 8.1, and 7.8 per 1,000 admissions, respectively) [10, 30].

This study also solicited the monthly fluctuation in the rate of HA-BSIs, but the trend was generally steady. The isolates' overall prevalence rate was generally higher than that reported from North America and Western Europe [28]. According to our study, the prevalence rate of HA-BSIs fluctuated over age categories. The pattern obtained could be interpreted by the strength of the immune system. The immunity starts low when the baby is born and then increases as the age increases to a certain point. After that, immunity declines as age advances, rendering the body more susceptible to infection. The exciting part came when the age and gender-specific prevalence rate was tested. It showed that the prevalence rate of HA-BSI was greater in males than in females in each age group. Moreover, it revealed that age significantly affects the prevalence rate among males more than females. The prevalence rate among females seems steady over different age categories. A study carried out in Thailand has pointed to the age-gender-specific incidence rate of HA-BSI, and it found that the incidence rate of HA-BSI was higher in males than in females (0.8 vs. 0.6 per 1,000 patient days, IRR 1.2; 95% CI; 1.13 to 1.30, *P* < 0.001) [31].

Among HA-BSI cases in this study, males (53.5%) were insignificantly more than females (46.5%) (*P* = 0.16). The overall prevalence rate of HA-BSIs is slightly higher among males than in females (8.5 vs. 9.2 per 1,000 admissions). About a quarter of the total number of cases fall in the age group (0–15) years, in which the prevalence rate is higher than the other age groups (10.0 per 1000 admissions). In a similar study carried out in the New York city, HA-BSI was significantly associated with males (OR = 1.25, *P* < 0.001) and with patients in age categories 65–84 and >85 (OR = 1.12 and 1.37, respectively) [32]. A Swedish study found that males constitute 59% of HA-BSI cases (*P* < 0.001) [33].

The HA-BSI acquired in the intensive care unit (ICU) was a significant consequence of critical illness (K. B. Laupland et al., 2006). In this study, HA-BSI was more prevalent among patients admitted to the ICU (43.5 per 1000 admissions) compared to other hospital wards. The obtained prevalence rate was comparable to that reported in Australia (52 per 1000 ICU admissions) [34, 35]. This finding was much lower than that in Egypt, where the prevalence rate of HA-BSI was 75 per 1000 ICU admissions [36]. In Belgium Ghent University Hospital, the prevalence of HA-BSI among patients older than 45 years was 27.7 per 1000 ICU admissions from 1992 to 2006 [37]. In our study, most HA-BSI cases were reported in the medical ward (42.37%). This finding was comparable to the result obtained in Qatar, where the percentage was 49.5%. Our finding, however, was considerably higher than the results obtained in similar studies in Saudi Arabia, where HA-BSI cases from medical wards constitute 14.6% of the total cases [11, 14, 15].

In a recent study, the ICU came second after the medical ward by a percentage of 24.1, less than the percentages obtained in Qatar and Saudi Arabia (33.3% and 53.2%, respectively). It has been found that about a quarter of HA-BSI cases were in pediatric patients. This finding does not support Qatari (3.8%) and Saudi Arabian (4.8%) studies. The results from pediatric wards in the Qatari study and the Saudi study are converging, indicating better infection control measures in this ward. The percentages of HA-BSI cases reported in surgical wards were 9.1 and 3.2 in Qatar and Saudi Arabia, respectively. In our study, the percentage reported in the surgical ward is 10.9%. Thus, it is highly comparable to studies in Saudi Arabia [15, 16, 18].

### 4.1. Limitations

This study has some limitations, and it is worthwhile to highlight here. First, a nonprobability sampling method (convenience sampling) was used to collect the data from one hospital; hence, the results cannot be generalized to the whole country. Second, the relatively small sample size may have also affected the power of the study to detect significant differences. Not all observations were statistically significant across categories in the data analysis. Finally, since the study was cross-sectional, the HA-BSI occurrence indices were limited to prevalence only over retrospective follow-up, which implied a lack of temporality and potentially reversed causality. Incidence parameters would have been better measures of HA-BSI occurrence because they establish temporality.

## 5. Conclusion

In summary, this hospital-based, cross-sectional study explored the variation in the prevalence of HA-BSI among admitted patients in a tertiary hospital in Oman over five years of retrospective follow-up. The study provides supportive evidence for a slow reduction in HA-BSI prevalence over age categories and years of follow-up. It depicts the variety of gender-specific CRC prevalence estimates over increasing age categories. The study calls for the timely formulation and adoption of national HA-BSI screening programs geared toward increasing awareness of HA-BSI and considering screening as primary prevention to respond to the increase in HA-BSI prevalence in Oman and other Arab countries.

## Figures and Tables

**Table 1:** Selected sociodemographic and clinical characteristics of HA-BSI cases among admitted patients, Oman.

Characteristics	Total (*N* = 139683)	HA-BSI cases (*N* = 1246)	Noncases (*N* = 138437)	*P* value
	*N* (%)	*N* (%)	*N* (%)	
Gender				0.16
Female	67860 (48.6)	580 (46.5)	67280 (48.6)	
Male	71823 (51.4)	666 (53.5)	71157 (51.4)	
Age				0.47
0 to 15	32435 (23.2)	325 (26.1)	32110 (23.2)	
16 to 25	9874 (7.1)	97 (7.8)	9777 (7.1)	
26 to 35	21264 (15.2)	154 (12.4)	21110 (15.2)	
36 to 45	18349 (13.1)	128 (10.3)	18221 (13.2)	
46 to 55	11995 (8.6)	107 (8.6)	11888 (8.6)	
56 to 65	16264 (11.6)	154 (12.4)	16110 (11.6)	
66 to 75	20300 (14.5)	190 (15.2)	20110 (14.5)	
76 to 85	9202 (6.6)	91 (7.3)	9111 (6.6)	
Governorate				0.27
Dhofar	14113 (10.1)	167 (13.4)	13946 (10.1)	
Dakhiliyah	19176 (13.7)	218 (17.5)	18958 (13.7)	
Sharqiyah	17560 (12.6)	192 (15.4)	17368 (12.5)	
Batinah	25881 (18.5)	256 (20.5)	25625 (18.5)	
Dhahirah	10297 (7.4)	79 (6.3)	10218 (7.4)	
Muscat	48466 (34.7)	301 (24.2)	48165 (34.8)	
Buraimi	4190 (3.0)	22 (1.8)	4168 (3.0)	
Year of admission				0.08
2015	30598 (21.9)	275 (22.1)	30323 (21.9)	
2016	26933 (19.3)	232 (18.6)	26701 (19.3)	
2017	26229 (18.8)	264 (21.2)	25965 (18.8)	
2018	27986 (20.0)	259 (20.8)	27727 (20.0)	
2019	27937 (20.0)	216 (17.3)	27721 (20.0)	

**Table 2:** Prevalence estimates of HA-BSI among admitted patients and in isolates, stratified by year of admission, Oman.

Year	Admissions	BSI patients	BSI isolates	Prev. ^a^per patients	Prev. ^a^per isolates
2015	30598	275	363	9.0 (8.0, 10.1)	11.9 (10.7, 13.1)
2016	26933	232	303	8.6 (7.7, 9.9)	11.3 (10.1, 12.6)
2017	26229	264	339	10.1 (8.9, 11.3)	12.9 (11.6, 14.4)
2018	27986	259	323	9.3 (8.2, 10.4)	11.5 (10.4, 12.9)
2019	27937	216	274	7.7 (6.8, 8.8)	9.8 (8.7, 11.0)
Total	139683	1246	1602	8.9 (8.4, 9.4)	11.5 (10.9, 12.0)

^a^Prevalence per 1,000 admission.

**Table 3:** Prevalence estimates of HA-BSI among admitted patients and in isolates, stratified by month of admission, Oman.

Month	Admissions	BSI Patients	BSI isolates	Prev. ^a^per patients	Prev. ^a^per isolates
January	13710	118	150	8.6 (7.2, 10.3)	10.9 (9.3, 12.8)
February	10868	96	128	8.8 (7.2, 10.8)	11.8 (9.9, 14.0)
March	12866	102	135	7.9 (6.5, 9.6)	10.5 (8.9, 12.4)
April	11169	95	121	8.5 (7.0, 10.4)	10.8 (9.1, 12.9)
May	12858	113	137	8.8 (7.3, 10.6)	10.7 (9.0, 12.6)
June	10798	103	141	9.5 (7.9, 11.6)	13.1 (11.1, 15.4)
July	12364	96	130	7.8 (6.4, 9.5)	10.5 (8.9, 12.5)
August	10112	111	136	11.0 (9.1, 13.2)	13.4 (11.4, 15.9)
September	12125	107	138	8.8 (7.3, 10.7)	11.4 (9.6, 13.4)
October	10192	96	129	9.4 (7.7, 11.5)	12.7 (10.7, 15.0)
November	12304	109	138	8.9 (7.3, 10.7)	11.2 (9.5, 13.2)
December	10317	100	119	9.7 (8.0, 11.8)	11.5 (9.6, 13.8)
Total	139683	1246	1602	8.9 (8.4, 9.4)	11.5 (10.9, 12.0)

**Table 4:** Age- and gender-specific prevalence estimates of HA-BSI among admitted patients, Oman.

Age categories	Gender categories	Total (*N*)	Cases (*N*)	Prev.^a^ (95% CI)
Overall	Total	139683	1246	8.9 (8.4, 9.4)
	Female	67860	580	8.5 (7.9, 9.3)
	Male	71823	666	9.3 (8.6, 10.0)

15 or less	Total	32435	325	10.0 (9.0, 11.2)
	Female	18039	115	6.4 (5.3, 7.6)
	Male	14396	210	14.6 (12.8, 16.7)

16 to 25	Total	9874	97	9.8 (8.1, 12.0)
	Female	4913	25	5.1 (3.4, 7.5)
	Male	4961	72	14.5 (11.5, 18.2)

26 to 35	Total	21264	154	7.2 (6.2, 8.5)
	Female	8656	47	5.4 (4.1, 7.2)
	Male	12608	107	8.5 (7.0, 10.2)

36 to 45	Total	18349	128	7.0 (5.9, 8.3)
	Female	6732	42	6.2 (4.6, 8.4)
	Male	11617	86	7.4 (6.0, 9.1)

46 to 55	Total	11995	107	8.9 (7.4, 10.8)
	Female	7810	56	7.2 (5.5, 9.3)
	Male	4185	51	12.2 (9.3, 16.0)

56 to 65	Total	16264	154	9.5 (8.1, 11.1)
	Female	8877	47	5.3 (4.0, 7.0)
	Male	7387	107	14.5 (12.0, 17.5)

66 to 75	Total	20300	190	9.4 (8.1, 10.8)
	Female	9993	63	6.3 (4.9, 8.1)
	Male	10307	127	12.3 (10.4, 14.6)

76 or more	Total	9202	91	9.9 (8.1, 12.1)
	Female	6643	48	7.2 (5.5, 9.6)
	Male	2559	43	16.8 (12.5, 22.6)

^a^Prevalence is per 1000 admission.

**Table 5:** Prevalence estimates of HA-BSI among admitted patients, stratified by admitting ward, Oman.

Admitting ward	Admissions	BSI patients	Prev. ^a^per patients
Medical	31221	528	16.9 (15.5, 18.4)
Surgical	35684	136	3.8 (3.2, 4.5)
Child health	31634	265	8.4 (7.4, 9.4)
ICU	6915	301	43.5 (39.0, 48.6)
OBGYN	34229	16	0.5 (0.3, 0.8)
Total	139683	1246	8.9 (8.4, 9.4)

^a^Prevalence per 1,000 admission.

**Table 6:** Prevalence estimates of HA-BSI among admitted patients, stratified by admitting ward, Oman.

Ward	Total	Medical	Surgical	Child health	ICU	OBGYN
		(*N* = 31221)	(*N* = 35684)	(*N* = 31634)	(*N* = 6915)	(*N* = 34229)
Year 2015						
Cases	275	105 (38.2)	32 (11.6)	80 (29.1)	57 (20.7)	1 (0.4)
Admissions prev. (95% CI)	30598	6697 (21.9)	7961 (26.0)	6654 (21.7)	1588 (5.2)	7698 (25.2)
		15.7 (13.0, 18.9)	4.0 (2.8, 5.7)	12.0 (9.7, 14.9)	35.9 (27.8, 46.2)	0.1 (0.0, 0.7)
Year 2016						
Cases	232	95 (40.9)	37 (15.9)	41 (17.7)	54 (23.3)	5 (2.2)
Admissions prev. (95% CI)	26933	6079 (22.6)	6738 (25.0)	5756 (21.4)	1407 (5.2)	6953 (25.8)
		15.6 (12.8, 19.1)	5.5 (4.0, 7.6)	7.1 (5.3, 9.6)	38.4 (29.5, 49.7)	0.7 (0.3, 1.7)
Year 2017						
Cases	264	121 (45.8)	18 (6.8)	50 (18.9)	74 (28.0)	1 (0.4)
Admissions prev. (95% CI)	26229	6053 (23.1)	6231 (23.8)	6647 (25.3)	1054 (4.0)	6244 (23.8)
		20.0 (16.8, 23.8)	2.9 (1.8, 4.6)	7.5 (5.7, 9.9)	70.2 (56.3, 87.2)	0.2 (0.0, 0.9)
Year 2018						
Cases	259	114 (44.0)	28 (10.8)	55 (21.2)	59 (22.8)	3 (1.2)
Admissions prev. (95% CI)	27986	6142 (21.9)	7627 (27.3)	6239 (22.3)	1487 (5.3)	6491 (23.2)
		18.6 (15.5, 22.2)	3.7 (2.5, 5.3)	8.8 (6.8, 11.5)	39.7 (30.9, 50.8)	0.5 (0.2, 1.4)
Year 2019						
Cases	216	93 (43.1)	21 (9.7)	39 (18.1)	57 (26.4)	6 (2.8)
Admissions prev. (95% CI)	27937	6250 (22.4)	7127 (25.5)	6338 (22.7)	1379 (4.9)	6843 (24.5)
		14.9 (12.2, 18.2)	2.9 (1.9, 4.5)	6.2 (4.5, 8.4)	41.3 (32.0, 53.2)	0.9 (0.4, 1.9)

**Table 7:** Governorate-specific prevalence estimates of HA-BSI among admitted patients, Oman.

Governorate	Admissions	BSI patients	Prev. ^a^ per patients
Dhofar	14113	167	11.8 (10.2, 13.8)
Dakhiliyah	19176	218	11.4 (10.0, 13.0)
Sharqiyah	17560	192	10.9 (9.5, 12.6)
Batinah	25881	256	9.9 (8.8, 11.2)
Dhahirah	10297	79	7.7 (6.2, 9.6)
Muscat	48466	301	6.2 (5.5, 7.0)
Buraimi	4190	22	5.3 (3.5, 7.9)
Total	139683	1246	8.9 (8.4, 9.4)

^a^Prevalence per 1,000 admission.

## Data Availability

The data used to support the findings of this study are available from the corresponding author upon request (ymfarsi@squ.edu.om).
